# Selections from a Clinical Lecture on Tumors

**Published:** 1871-01

**Authors:** W. T. Briggs


### Selections.

### SELECTIONS FROM A CLINICAL LECTURE ON TUMORS.

BY PROF. W. T. BRIGGS.

There is, probably, no class of disease of more interest to the practical surgeon than that of tumors. Their frequency, their variety, and their relations, render their consideration of the first importance.

John Hunter long since defined a tumor to be a circumscribed substance, produced by disease, different, in its nature and consistence, from the surrounding parts. We mean, when we use the term tumor, a circumscribed mass growing in some tissue or organ of the body, depending on some change in nutrition, having an inherent power of growth, and departing widely from the normal form of the part upon which it is situated. They are always the result of perverted growth and nutrition.

Tumors are divisable into two chief groups—benign and malignant. The distinctive characters of the benign, or innocent, tumors, are: 1st, that they are formed of structures very much like those of the natural parts of the body. 2d, that they do not exclude, or replace, the structure of the part in which they are situated. 3d, that the degeneration and disease that may occur in them, are not prone to extend beyond them. 4th, that they very seldom multiply or propagate themselves. 5th, that they are generally single, and, if multiple, they are found in the same part or tissue. 6th, that when removed, they are not likely to recur.

The characters of malignant tumors are; 1st, that they are formed of structures very unlike the natural tissues of the body. 2d, that they are infiltrated into the tissues of the part, and exclude those tissues. 3d, that when they ulcerate, they do not confine their destructive effects to themselves, but extend beyond their boundary, and invade every structure in the neighborhood. 4th, that they not only enlarge, but multiply and propagate themselves. 5th, that in their multiplication and ulceration there is hardly a tissue or organ they may not invade. 6th, that when removed, they invariably return.

These distinctive characters of tumors should be well remembered; for, while it often happens that we may be unable to tell what particular class a tumor belongs to, we can, by closer examination, almost always, say whether they belong to the group of benign or malignant tumors.

Again, the group of innocent tumors may be divided into the cystic and solid. The cysts are of two kinds—1st, simple, or barren; 2d, compound, or proliferous.

The simple cyst consist of a sac, or bag, filled with a fluid, either its product by secretion, or by endogenous growth. They may contain serum, blood, synovia, oil, or seminal fluid, and are named according to their contents.

The proliferous are those which produce organized structures from their inner wall, either secondary cyst, single or multiple, or vascular growth, such as glandular, cutaneous, or dentigerous products.

The solid tumors are formed of tissues precisely like the natural tissues of the body, and are named, according to their structure, fatty, fibro-cellular, fibrous, cartilaginous, osseous, &c., &c.

Tumors may be situated in any part of the body. Malignant growths are most generally seated in the glandular organs; benign in the skin and cellulo-adipose tissue. They assume a variety of forms—globular, oval, flat, pendulous, pyriform, or pediculated. They vary exceedingly in their volume—sometimes not larger than a millet seed, and again as large as the patient's body.

As we have already seen, they differ very much in their consistence.

Their mobility is dependent on their nature, situation, &c. Some are never movable, others always, whatever may be their age, volume, or situation. Usually, when small, they give rise to no uneasiness in the part where located; but as they grow, and become large, they encroach upon and irritate the surrounding tissues; they cause absorption by their pressure, and often, too, seriously interfere with function.

Having an inherent growth, almost entirely independent of the body, it is not astonishing that medicines have but ittle effect in removing, or even arresting their progress.

They very rarely disappear spontaneously. Sometimes, however, they disappear by absorption, or ulceration, or gangrene. It is only by surgical procedure that they can be got rid of.

The operation for their removal is sometimes so simple and insignificant, that the merest tyro may perform it; at others, the most intimate anatomical knowledge, and the highest surgical skill, is requisite for their safe extirpation.—*Nashville Medical Journal.*

